# Supplementary material for: Use of the journal impact factor for assessing individual articles: Statistically flawed or not?
Source: F1000Res. 2021 Mar 1;9:366. Originally published 2020 May 14. [Version 2] doi: 10.12688/f1000research.23418.2 (PMC7974631; doi:10.12688/f1000research.23418.2)
Supplement: Peer review history

# Peer review: Reviewer response for version 2

**DOI:** 10.5256/f1000research.54872.r80407

**Published:** 2021-03-18

**Authors:** Stephen Curry

**Affiliations:** 1 Department of Life Sciences, Imperial College London, London, UK

I thank the authors for their response to my review and the amendments that they have incorporated into the revised paper. This helps to clarify a number of aspects of their argument. 

 We seem to remain on opposite sides of a philosophical debate as to the meaning and impact of their theoretical analysis. Nevertheless, I appreciate the care they have brought to their revision.

 My concern remains with questions about how this theoretical analysis might shape the real world. If I were to ask for one further minor revision it would be to mention in the abstract an important caveat that is found within the body of the paper: "Our analysis is of a theoretical nature, and it, therefore, does not make clear whether *in practice* the use of the IF for assessing individual articles is to be recommended or not and whether *in practice* the IF is more or less accurate than citations."

Is the work clearly and accurately presented and does it cite the current literature?

Yes

If applicable, is the statistical analysis and its interpretation appropriate?

Yes

Are all the source data underlying the results available to ensure full reproducibility?

Yes

Is the study design appropriate and is the work technically sound?

Yes

Are the conclusions drawn adequately supported by the results?

Partly

Are sufficient details of methods and analysis provided to allow replication by others?

Yes

Reviewer Expertise:

Structural biology; research assessment; research culture

I confirm that I have read this submission and believe that I have an appropriate level of expertise to confirm that it is of an acceptable scientific standard.

# Peer review: Reviewer response for version 1

**DOI:** 10.5256/f1000research.25847.r63448

**Published:** 2020-05-29

**Authors:** Stephen Curry

**Affiliations:** 1 Department of Life Sciences, Imperial College London, London, UK

*In the interests of full disclosure, I want to declare that I am the chair of the steering group of DORA and the author of the blogpost ‘Sick of Impact Factors’, both of which are subject to critique by the authors of this manuscript. These potential conflicts of interest should be borne in mind when reading this review.*

 This manuscript presents a theoretical model to contest the view that statistical considerations, primarily the inappropriateness of a single indicator – the journal impact factor (JIF) – to characterise the skewed citation distributions found in all journals, should not be grounds for denying it a role in the assessment of individual articles. The rationale for this work is presented carefully and the theoretical model that forms the centre-piece of the manuscript is clearly laid out. The authors take some pains to acknowledge the simplified nature of this exercise and the wider (and in my view more substantive) debate surrounding the problematic nature of undue reliance on journal impact factors in research assessment. On the whole, this manuscript is an interesting and thoughtful contribution to an important debate and I enjoyed reading it. However, I think there are still some serious problems, both technical and rhetorical, that the authors should address.

 First, the problem being addressed is extremely narrowly framed. To a degree, the authors have been admirably precise in outlining the very particular objection that they seek to address. This is centred on the particular question of whether the skewness of journal citation distributions means that use of the JIF or the paper citation count is a better indicator of the value of an individual article. 

 However, this very narrow focus comes at the cost of discounting many additional arguments against the use of aggregate indicators like the JIF to assess individual papers. These include problems – many of which are statistical in nature – with the definitions of citable items, the JIF’s short time window, the lack of transparency of the underlying data, the possible manipulation of the JIF, and the performative impact of explicit reliance on JIFs in assessment criteria. Although these problems are discussed briefly at the beginning of section 2 of the paper, they are downplayed in the authors’ subsequent survey of the literature on statistical objections to the JIF. To take one example, the authors quote a single phrase from the DORA declaration ( https://sfdora.org/read/) but make no mention of the richer argument that is presented there describing the problematic nature of the JIF. I accept that the skewness of this discussion is partially justified by the authors’ desire to focus their critique, but that focus needs to be maintained throughout. In the discussion, the authors claim that their analysis shows that “commonly used statistical arguments” against the use of the JIF are incorrect, but in fact, they have only addressed one particular statistical (or technical) argument. I think more precise phrasing is warranted. A reminder to the reader in the discussion of the unaddressed technical problems with the JIF (which are not taken into account in the model) would be also helpful. 

 Second, there is to my mind a weakness in the argument constructed from the simple example presented in section 3.2. This theoretical model shows (for the synthetic data relating to 200 papers presented in Tables 2 and 3) that it is possible to imagine scenarios where either reliance on the JIF or paper citation counts could give more accurate selections of groups of high-value papers. While this may be the case, this type of selection (of groups of papers) does not map onto any real-world exercises in research assessment. Further, the claim following from this argument that “the number of citations of an article is not necessarily a more accurate indicator of the value of the article than the IF of the journal in which the article has appeared,” is correct only insofar as the method allows the calculation of a probability that one or the other selection method will identify a high-value article, a probability that can only be determined for synthetic data. The probabilistic nature of the authors’ claim should be given more emphasis.  

 This weakness of this argument is not resolved in the more sophisticated mathematical modelling presented in section 5. Although the authors are at pains to point out the theoretical nature of their argument and the fact that it presents a simplified view of reality, as they themselves concede, there is no visible route to testing their hypothetical model with real data. As an academic exercise, there is some merit in using purely theoretical approaches to think through a problem, but ultimately theoretical models should give rise to real-world predictions or applications. It is somewhat telling that, although a previous version of this manuscript was posted to the arXiv in 2017, in the intervening three years the authors have made no further progress in testing their ideas with real data. This is likely an indication of the immense difficulty in producing accurate estimates for the key parameters of the model, sigma-r-squared (the accuracy of journal peer review) and sigma-c-squared (the accuracy of citations as an indicator of value). The manuscript discusses how “empirical follow-up research” might be conducted but the ideas presented are speculative or confine themselves to the pursuit of more sophisticated simulations. In the absence of a test on real data – or a clear pathway to such a test – the conclusions drawn must remain hypothetical and unconvincing. 

 There is some implicit acknowledgement of these limitations in the authors’ careful use of language, especially perhaps in the “need not” of their title and the double-negative construction in the abstract: “Our analysis show that these arguments do not support the conclusion that the impact factor should not be used for assessing individual articles.” 

 However, I don’t think these go far enough. I have found myself asking whether the authors have fully considered the rhetorical impact of their title and summary, which may well be the only sections of this paper read by university research managers. Obviously, it is the responsibility of readers to read in full, but the Leiden Manifesto ( https://www.nature.com/news/bibliometrics-the-leiden-manifesto-for-research-metrics-1.17351), of which Waltman is a co-author, urges us all to act responsibly in thinking about and using metrics. In the spirit of the manifesto, I would strongly suggest that the authors modify their title and abstract to make it clear that they have no way to apply the theoretical analysis presented in their paper to decisions that may impact the careers of real people and the conduct of research.

Is the work clearly and accurately presented and does it cite the current literature?

Yes

If applicable, is the statistical analysis and its interpretation appropriate?

Yes

Are all the source data underlying the results available to ensure full reproducibility?

Yes

Is the study design appropriate and is the work technically sound?

Yes

Are the conclusions drawn adequately supported by the results?

Partly

Are sufficient details of methods and analysis provided to allow replication by others?

Yes

Reviewer Expertise:

Structural biology; research assessment; research culture

I confirm that I have read this submission and believe that I have an appropriate level of expertise to confirm that it is of an acceptable scientific standard, however I have significant reservations, as outlined above.

# Response

**Published:** 2021-02-20

**Authors:** Ludo Waltman

**Affiliations:** Leiden University, The Netherlands

*Thank you for reviewing our paper. We appreciate your in-depth feedback. Please find below a point-by-point response.*

 In the interests of full disclosure, I want to declare that I am the chair of the steering group of DORA and the author of the blogpost ‘Sick of Impact Factors’, both of which are subject to critique by the authors of this manuscript. These potential conflicts of interest should be borne in mind when reading this review.

 This manuscript presents a theoretical model to contest the view that statistical considerations, primarily the inappropriateness of a single indicator – the journal impact factor (JIF) – to characterise the skewed citation distributions found in all journals, should not be grounds for denying it a role in the assessment of individual articles. The rationale for this work is presented carefully and the theoretical model that forms the centre-piece of the manuscript is clearly laid out. The authors take some pains to acknowledge the simplified nature of this exercise and the wider (and in my view more substantive) debate surrounding the problematic nature of undue reliance on journal impact factors in research assessment. On the whole, this manuscript is an interesting and thoughtful contribution to an important debate and I enjoyed reading it. However, I think there are still some serious problems, both technical and rhetorical, that the authors should address.

 First, the problem being addressed is extremely narrowly framed. To a degree, the authors have been admirably precise in outlining the very particular objection that they seek to address. This is centred on the particular question of whether the skewness of journal citation distributions means that use of the JIF or the paper citation count is a better indicator of the value of an individual article.

 However, this very narrow focus comes at the cost of discounting many additional arguments against the use of aggregate indicators like the JIF to assess individual papers. These include problems – many of which are statistical in nature – with the definitions of citable items, the JIF’s short time window, the lack of transparency of the underlying data, the possible manipulation of the JIF, and the performative impact of explicit reliance on JIFs in assessment criteria. Although these problems are discussed briefly at the beginning of section 2 of the paper, they are downplayed in the authors’ subsequent survey of the literature on statistical objections to the JIF. To take one example, the authors quote a single phrase from the DORA declaration (https://sfdora.org/read/) but make no mention of the richer argument that is presented there describing the problematic nature of the JIF. I accept that the skewness of this discussion is partially justified by the authors’ desire to focus their critique, but that focus needs to be maintained throughout. In the discussion, the authors claim that their analysis shows that “commonly used statistical arguments” against the use of the JIF are incorrect, but in fact, they have only addressed one particular statistical (or technical) argument. I think more precise phrasing is warranted. A reminder to the reader in the discussion of the unaddressed technical problems with the JIF (which are not taken into account in the model) would be also helpful.

*In the context of the IF, there are two types of statistical objections that are commonly raised. First, there are statistical objections that apply to the IF in general, irrespective of the level at which the IF is used (i.e., at the journal level or at the article level). Second, there are statistical objections that are specifically about the use of the IF, and of journal-level indicators more generally, at the article level. Our paper is about the latter objections, not about the former ones. As our literature review shows, the latter objections play a prominent role in the debate about the IF, and they therefore deserve close attention. The latter objections are also more fundamental than the former ones, since the former objections could be addressed by modifying the way in which the IF is calculated, which is not possible for the latter objections.*

*Throughout our paper, we point out that our focus is on statistical objections against the use of the IF at the level of individual articles, not on other statistical objections against the IF. In our revised paper, we have made a number of changes in the introductory and concluding sections to emphasize this point even more. In addition, as suggested by the reviewer, in our revised paper we are more explicit about the fact that there are also other statistical objections against the IF, which we do not consider in our paper.*

 Second, there is to my mind a weakness in the argument constructed from the simple example presented in section 3.2. This theoretical model shows (for the synthetic data relating to 200 papers presented in Tables 2 and 3) that it is possible to imagine scenarios where either reliance on the JIF or paper citation counts could give more accurate selections of groups of high-value papers. While this may be the case, this type of selection (of groups of papers) does not map onto any real-world exercises in research assessment. Further, the claim following from this argument that “the number of citations of an article is not necessarily a more accurate indicator of the value of the article than the IF of the journal in which the article has appeared,” is correct only insofar as the method allows the calculation of a probability that one or the other selection method will identify a high-value article, a probability that can only be determined for synthetic data. The probabilistic nature of the authors’ claim should be given more emphasis.

*In our revised paper, we have added a sentence in which we explicitly indicate the probabilistic nature of our example. However, we do not see this probabilistic nature as a weakness. Indicators provide only approximate information (which is why we call them indicators rather than metrics). Sometimes the information provided by an indicator is accurate, while in other cases it is not. This applies both to journal-level indicators and to article-level indicators. The probabilistic nature of our example is a reflection of the probabilistic nature of indicators.*

 This weakness of this argument is not resolved in the more sophisticated mathematical modelling presented in section 5. Although the authors are at pains to point out the theoretical nature of their argument and the fact that it presents a simplified view of reality, as they themselves concede, there is no visible route to testing their hypothetical model with real data. As an academic exercise, there is some merit in using purely theoretical approaches to think through a problem, but ultimately theoretical models should give rise to real-world predictions or applications. It is somewhat telling that, although a previous version of this manuscript was posted to the arXiv in 2017, in the intervening three years the authors have made no further progress in testing their ideas with real data. This is likely an indication of the immense difficulty in producing accurate estimates for the key parameters of the model, sigma-r-squared (the accuracy of journal peer review) and sigma-c-squared (the accuracy of citations as an indicator of value). The manuscript discusses how “empirical follow-up research” might be conducted but the ideas presented are speculative or confine themselves to the pursuit of more sophisticated simulations. In the absence of a test on real data – or a clear pathway to such a test – the conclusions drawn must remain hypothetical and unconvincing.

*We do not agree that our conclusions are unconvincing. We conclude that the use of journal-level indicators such as the IF at the level of individual articles need not be statistically wrong. This conclusion follows logically from our analysis. If the reviewer is not convinced that this conclusion is justified, the reviewer should point to a logical flaw in our analysis.*

*Importantly, we do not conclude that journal-level indicators are preferable over article-level indicators. Our analysis does not support such a conclusion. Those who want to argue that journal-level indicators are preferable over article-level indicators indeed need to provide additional empirical evidence. Likewise, those who want to argue that article-level indicators are preferable over journal-level indicators also need to provide additional empirical evidence. The burden of proof is not on us, but on those who want to argue in favor of either journal-level or article-level indicators. Regarding the choice between the two types of indicators, we consider ourselves to be agnostic. In fact, depending on the context, we think it may not even be desirable to choose between the two types of indicators and it may in fact be better to combine the use of the different types of indicators, so that a more complete picture is obtained.*

 There is some implicit acknowledgement of these limitations in the authors’ careful use of language, especially perhaps in the “need not” of their title and the double-negative construction in the abstract: “Our analysis show that these arguments do not support the conclusion that the impact factor should not be used for assessing individual articles.”

 However, I don’t think these go far enough. I have found myself asking whether the authors have fully considered the rhetorical impact of their title and summary, which may well be the only sections of this paper read by university research managers. Obviously, it is the responsibility of readers to read in full, but the Leiden Manifesto (https://www.nature.com/news/bibliometrics-the-leiden-manifesto-for-research-metrics-1.17351), of which Waltman is a co-author, urges us all to act responsibly in thinking about and using metrics. In the spirit of the manifesto, I would strongly suggest that the authors modify their title and abstract to make it clear that they have no way to apply the theoretical analysis presented in their paper to decisions that may impact the careers of real people and the conduct of research.

*We do not agree with the reviewer that we “have no way to apply the theoretical analysis presented in (our) paper to decisions that may impact the careers of real people and the conduct of research”. Our analysis shows that article-level indicators are not necessarily preferable over journal-level indicators, and this point has important real-world consequences. Recent developments at the Medical Faculty of the University of Bern illustrate this. After signing DORA, this institution decided to replace the IF by the relative citation ratio (RCR; Steck, Stalder, & Egger, 2020) to support decision making about promotions. The IF is a relatively transparent indicator, enabling anyone who uses the indicator to reflect on its pros and cons and to discuss what kinds of conclusions can and cannot be drawn from the indicator. The RCR is a highly complex black-box indicator, making it much more difficult for the typical user of the indicator to reflect on its pros and cons and to discuss how the indicator can best be interpreted. In addition, just like the IF calculation, the calculation of the RCR has been subject to substantial criticism. From the viewpoint of responsible research assessment, we believe that the use of a relatively transparent journal-level indicator may be preferable over the use of a non-transparent article-level indicator. This example, which we briefly discuss in footnote 7 in our revised paper, illustrates the danger of dogmatically insisting that article-level indicators are preferable over journal-level indicators.*

*Despite our disagreement with the reviewer, we have decided to make a (small) change to the title of our paper. The title has been changed from ‘Use of the journal impact factor for assessing individual articles need not be statistically wrong’ to ‘Use of the journal impact factor for assessing individual articles: Statistically flawed or not?’. This will hopefully stimulate readers to carefully reflect on the use of the IF for assessing individual articles and to neither reject nor accept this practice in an uncritical manner, but to engage in a more meaningful discussion on pros and cons of the use of journal-level indicators. We have also made some (small) changes to the abstract of our paper.*

# Peer review: Reviewer response for version 1

**DOI:** 10.5256/f1000research.25847.r63446

**Published:** 2020-05-15

**Authors:** Mike Thelwall

**Affiliations:** 1 Statistical Cybermetrics Research Group, University of Wolverhampton, Wolverhampton, UK

This paper uses a mix of conceptual analysis, simplified examples, and simulations to argue that using journal Impact Factors (IFs) is not from a statistical perspective inferior to using citation counts for the task of identifying high-value articles. The argument revolves around the key point that neither the citation count of the article nor the IF of its journal measures the value of the article: both are (approximate) indicators of its value, however the concept of value is interpreted. Once this point is accepted, which I think it must be, then this article proves that on a purely statistical basis it is impossible to say whether citation counts or IFs are the best indicators of value for individual articles.

 The point that the authors make that both IFs and citations are indicators of something that we cannot measure is a key one that needs to be understood by anyone using citation-based indicators for evaluations. I suspect that this will make sense to many authors that value some journals above others but recognize that even these occasionally publish poor articles.

 As the authors make clear, they are not arguing in favour of or against the use of IFs for article-level evaluations, they are only showing that one of the arguments against the use of IFs is not correct. I think that this paper makes a positive contribution by shifting the debate to non-statistical issues when considering the value of IFs (but see below).

 I have one minor quibble: since the IF is calculated using methods that do not take into account the skewing of citation counts, I think it is reasonable to call the IF statistically illiterate, even though the authors have demonstrated that it is not statistically illiterate to use the IF for identifying high value articles.

Is the work clearly and accurately presented and does it cite the current literature?

Yes

If applicable, is the statistical analysis and its interpretation appropriate?

Yes

Are all the source data underlying the results available to ensure full reproducibility?

No source data required

Is the study design appropriate and is the work technically sound?

Yes

Are the conclusions drawn adequately supported by the results?

Yes

Are sufficient details of methods and analysis provided to allow replication by others?

Yes

Reviewer Expertise:

Scientometrics

I confirm that I have read this submission and believe that I have an appropriate level of expertise to confirm that it is of an acceptable scientific standard.

# Response

**Published:** 2021-02-20

**Authors:** Ludo Waltman

**Affiliations:** Leiden University, The Netherlands

*Thank you for reviewing our paper. Please find below a point-by-point response.*

 This paper uses a mix of conceptual analysis, simplified examples, and simulations to argue that using journal Impact Factors (IFs) is not from a statistical perspective inferior to using citation counts for the task of identifying high-value articles. The argument revolves around the key point that neither the citation count of the article nor the IF of its journal measures the value of the article: both are (approximate) indicators of its value, however the concept of value is interpreted. Once this point is accepted, which I think it must be, then this article proves that on a purely statistical basis it is impossible to say whether citation counts or IFs are the best indicators of value for individual articles.

 The point that the authors make that both IFs and citations are indicators of something that we cannot measure is a key one that needs to be understood by anyone using citation-based indicators for evaluations. I suspect that this will make sense to many authors that value some journals above others but recognize that even these occasionally publish poor articles.

 As the authors make clear, they are not arguing in favour of or against the use of IFs for article-level evaluations, they are only showing that one of the arguments against the use of IFs is not correct. I think that this paper makes a positive contribution by shifting the debate to non-statistical issues when considering the value of IFs (but see below).

 I have one minor quibble: since the IF is calculated using methods that do not take into account the skewing of citation counts, I think it is reasonable to call the IF statistically illiterate, even though the authors have demonstrated that it is not statistically illiterate to use the IF for identifying high value articles.

*In our view, the use of a journal-level indicator, such as the IF, at the level of individual articles is not statically illiterate. We do concede that there could be reasons for questioning some of the statistical properties of the IF. To better indicate our position on this issue, we have rephrased the sentence on statistical illiteracy in the concluding section of our paper.*
